# *In silico* definition of new ligninolytic peroxidase sub-classes in fungi and putative relation to fungal life style

**DOI:** 10.1038/s41598-019-56774-4

**Published:** 2019-12-30

**Authors:** Catherine Mathé, Nizar Fawal, Christophe Roux, Christophe Dunand

**Affiliations:** Laboratoire de Recherche en Sciences Végétales, Université de Toulouse, CNRS, UPS, Toulouse, France

**Keywords:** Evolution, Bioinformatics

## Abstract

Ligninolytic peroxidases are microbial enzymes involved in depolymerisation of lignin, a plant cell wall polymer found in land plants. Among fungi, only Dikarya were found to degrade lignin. The increase of available fungal genomes allows performing an expert annotation of lignin-degrading peroxidase encoding sequences with a particular focus on Class II peroxidases (CII Prx). In addition to the previously described LiP, MnP and VP classes, based on sequence similarity, six new sub-classes have been defined: three found in plant pathogen ascomycetes and three in basidiomycetes. The presence of CII Prxs could be related to fungal life style. Typically, necrotrophic or hemibiotrophic fungi, either ascomycetes or basidiomycetes, possess CII Prxs while symbiotic, endophytic or biotrophic fungi do not. CII Prxs from ascomycetes are rarely subjected to duplications unlike those from basidiomycetes, which can form large recent duplicated families. Even if these CII Prxs classes form two well distinct clusters with divergent gene structures (intron numbers and positions), they share the same key catalytic residues suggesting that they evolved independently from similar ancestral sequences with few or no introns. The lack of CII Prxs encoding sequences in early diverging fungi, together with the absence of duplicated class I peroxidase (CcP) in fungi containing CII Prxs, suggests the potential emergence of an ancestral CII Prx sequence from the duplicated CcP after the separation between ascomycetes and basidiomycetes. As some ascomycetes and basidiomycetes did not possess CII Prx, late gene loss could have occurred.

## Introduction

Plant cell walls represent the major components of plant biomass. As both a physical barrier and a dynamic compartment, they play important roles in the development of plants and their interactions with the environment, but also in ecology as they are major components of the carbon cycle. Th primary cell wall mainly consists of celluloses, hemicelluloses, pectins and a minority of structural and enzymatic proteins which confer growth abilities to the plant. The secondary cell wall mostly contains cellulose and lignin, a complex polyphenolic polymer responsible for land plant rigidity and associated with the vessels appearance, with notable difference in composition between softwood (coniferous) and hardwood (deciduous). Altogether, they form intricate macromolecular networks that are synthesized by complex processes. Lignocellulosic cell walls are carbon sources which can therefore be degraded by micro-organisms such as some bacteria and more efficiently by certain fungi^1,2^. Plant cell walls are decayed primarily by wood and litter decomposers that easily digest cellulosic compounds due to enzymatic activities. However, the accessibility of these compounds is restricted by the presence of the more resistant lignin polymers. Therefore, efficient lignin depolymerization capacities are necessary and are developed by wood-decaying basidiomycetes^3^. In a recent review^2^, ligninolytic enzymes were inventoried into three categories: phenol oxidases (or laccases), class II peroxidases (CII Prxs thereafter, also known as POD) and Dye-decolorizing peroxidases. These last ones (called DyP-type peroxidases) are more reported in bacteria than in fungi^4^, and little is known about their physiological role. Historically, wood-decaying fungi have been characterized as white-rot (WR) or brown-rot (BR) fungi due to the color of remaining woody fragments. WR fungi can either degrade both lignin and polysaccharides (simultaneous decay) or preferentially remove lignin, leaving most of the cell wall polysaccharides unaffected (selective decay). As an example, *Phanerochaete chrysosporium* is a very efficient wood decomposer; that can simultaneously degrade lignin and cellulose^5–7^. A closely related species, *Ceriporiopsis subvermispora*, preferentially depolymerizes lignin with reduced cellulose degradation^8^. Genomes of several WR fungi have been screened for the presence of ligninolytic peroxidases such as *Agaricus bisporus* or *Pleurotus ostreatus*, which possess limited copies of CII Prx^9,10^. BR fungi, like *Postia placenta* and *Serpula lacrymans*, are mainly capable of pectin, hemicellulose and cellulose break-down but have only few or no lignin-degrading enzymes such as the CII Prx^11–13^. BR fungi preferentially attack softwoods i.e. gymnosperm species. The lack of CII Prx is also observed in the ectomycorrhizal (ECM) fungus *Laccaria bicolor*^14^ as well as others ECM^15^ suggesting that the reduced number or absence of CII Prx isoforms could be correlated with the mode of carbon acquisition, and then the type of interaction with the host plants. The hypothesis is that the loss of CII Prx in ECM was initiated in their saprotroph ancestor and therefore preceded the evolution of the ECM habit^15^. It is also important to note that a no ligninolytic fungi belongs to rhizosphere microbial communities containing ligninolytic fungi which could act synergistically. Phylogenetic analysis of CII Prx from Agaromycetes and from Hymenochaetales suggested that lignin degradation by WR fungi is strongly correlated with the presence of ligninolytic peroxidases and could not only be performed by laccases, the other ligninolytic enzyme group^16,17^. It was even proposed to limit the “white rot” term to fungi that possess CII Prx^18^, as many fungi are intermediate between WR and BR in wood decay mechanism^18–20^.

CII Prxs belong to the non-animal haem peroxidases superfamily together with the intracellular class I peroxidases (CI Prxs) and secretory plant class III peroxidases (CIII Prxs)^21^. The three classes CI, CII and CIII Prx are all characterized by a prosthetic group and 10 conserved α-helices^22^. In these classes, various substrates can be oxidized with hydrogen peroxide (H_2_O_2_) as the electron acceptor and reduced to water. The amino acids necessary for haem binding and to the H_2_O_2_ access channel are conserved between the various peroxidase classes. The CII Prxs, only detected in fungi, are extracellular peroxidases that were initially subdivided into 3 groups based on their catalytic properties: lignin (LiP), manganese (MnP) and versatile (VP) peroxidases^23^. Additional genomic data allowed the identification of a fourth group found in basidiomycetes, called “generic peroxidases” (GP)^24^. Three residues (Glu35, Glu39 and Asp179 in the mature PcMnP01) have been identified as implicated in the manganese-dependent activity^25^. These residues are well conserved within the MnP and VP proteins. VP were originated thanks to the appearance of an exposed tryptophan, implied in oxidation of nonphenolic lignin. Later, the loss of the Mn^2+^-binding site led to the generation of LiP proteins, which possess higher catalytic efficiency^26^.

Many investigations were done these last years regarding the evolution and distribution of CII Prxs among fungal genomes^15,24,27,28^. The resulting hypothesis is that CII Prx exists in the ancestor of Auriculariales and Agaricomycetes among basidiomycetes, with subsequent gene expansion of this family in saprotrophic fungal species involved in wood decay, degrading lignin to obtain their organic carbon sources. These works also indicated that ectomycorrhizal fungal species that later arose from independent lineages similarly present a loss of CII Prx gene as a convergent genome adaptation for symbiosis, thus limiting host immunity responses. Finally, CII Prx could also be a means for pathogenic species to degrade host cell wall for penetration. In ascomycetes, putative CII Prxs have been neither described, nor included in the four previous CII Prx families. Ascomycetes have limited ability to degrade lignin and are mostly responsible for soft rot decay in wet environment^29^.

Wood decay, and particularly lignin degradation by fungal laccases and peroxidases has a major ecological incidence on the carbon cycle^30^ and presents industrial interests. However automatic genome annotation has been demonstrated to produce increased mis-predictions in the case of multigenic families and genes containing numerous exons and very short intron/exon sizes^31^. We mined publicly available basidiomycete and ascomycete genomes to perform an expert annotation of the different Prxs. We set up a protocol coupling automatic and manual annotation for an exhaustive and quality annotation in order to analyze the evolution process of this complex family.

## Material and Methods

### Data mining and annotation with Scipio

An exhaustive data mining procedure was performed on the fungal genomes available from BROAD institute, JGI and NCBI to extract all the available ligninase sequences. All sequences used in this study have been annotated by an expert process to discard prediction errors subsequent to automatic annotations. First, a search by keyword and profile number has been done on different databases. These searches have been completed by homology searches against predicted proteomes (BLASTP), transcriptomes (TBLASTN) or whole genomes using well known ligninase sequences from Phanerochaetes. This data mining allowed the creation of initial batches corresponding to the different protein families. Then, a manual and individual curation of each predicted sequence was performed: gene structure (intron number and position), length and the presence of key residues were mandatory controls. When available mainly from NCBI, EST sequences have also been used to confirm annotations. These protein batches were then used to precisely determine the corresponding chromosome positions, gene structures and coding DNA sequences (CDS) with a protocol based on Scipio^32^. New paralogs, not initially annotated, were found using this procedure. We ended up with 267 sequences from Basidiomycota and 99 from Ascomycota.

### Phylogenetic analysis

All protein sequences used for the *in silico* analyses are available from the RedoxiBase database (http://peroxibase.toulouse.inra.fr)^33,34^. First, protein sequences were aligned using PRANK^35^ with default parameters. Then phylogenies were estimated by maximum likelihood using RaxML (version 8.1.5)^36^, under the PROTGAMMAWAG model, as the substitution model determined by protTest^37^ was WAG^38^ and a gamma distribution (4 discrete categories of sites and an estimated alpha parameter). Finally, the trees were edited and analyzed using iTOL (https://itol.embl.de/).

### Gene structure analysis

The intron/exon coordinates together with the corresponding genomic sequences of all identified genes were determined with Scipio^32^, with maximal intron size set to 1000 nt, and minimum percent identity set to 30%. The intron/exon conservation within the different families was verified with CIWOG^39^ and GECA^40^. They both analyzed the evolution or conservation of introns between paralogs as well as between species.

Intron size changes were visualized through the graphical representation provided by GECA.

### Conserved common introns analysis

Gene structure and common introns (or cintrons) were analyzed from all fungi sequences. First, the protein alignment generated with MAFFT^41^ was completed with the identification of common introns in the corresponding genes with CIWOG. Cintrons were extracted from the CIWOG database and only those present in one or more sub-classes with a conservation rate higher than 50% were considered as conserved. Finally, the sequences were placed in order of appearance in the phylogenetic tree and the conserved cintrons were highlighted for each sequence.

### Duplication analysis

In order to test whether the presence of transposable elements can explain a high duplication rate, RepeatMasker^42^ version 4.0.3 (with fungi specified as “species”) was run on all analyzed Basidiomycete genomes. No correlation can be made between the number of paralogs in an organism and the number of repeated sequences. Deeper analysis of repeated sequences positions was conducted for *Tramete versicolor* and *Galerina marginata* genomes (which possess the highest number of CII Prxs): neither transposable elements nor other repeated sequences were systematically detected nearby to a gene copy.

### New PROSITE profiles design and WebLogo

Using a global phylogenetic analysis, different protein clusters have been defined to update the existing PROSITE profiles^33^ and to design new specific profiles using the silenced residues. These profiles were built from full length alignments of each protein cluster. First, all the sequences from the different protein clusters were aligned with MAFFT. The sequence alignment was split into several sub-alignments according to the cluster definitions. Each cluster alignment contains an annotation line where residues conserved in the whole family are tagged. This annotation line is used to downweight family-conserved columns during the profile construction; therefore only cluster specific residues are taken into consideration. The reliability of each cluster is supported by both the analysis of the gene structures and the presence/absence of the key residues specific to the well described LiP, MnP and VP families. Furthermore, graphical sequence logos were created for each group with Weblogo3^43^ and aligned manually with the others in order to identify the amino acids conserved between the sub-classes.

## Results and Discussion

### Definition of new sub-classes of ligninases

**Figure 1:**
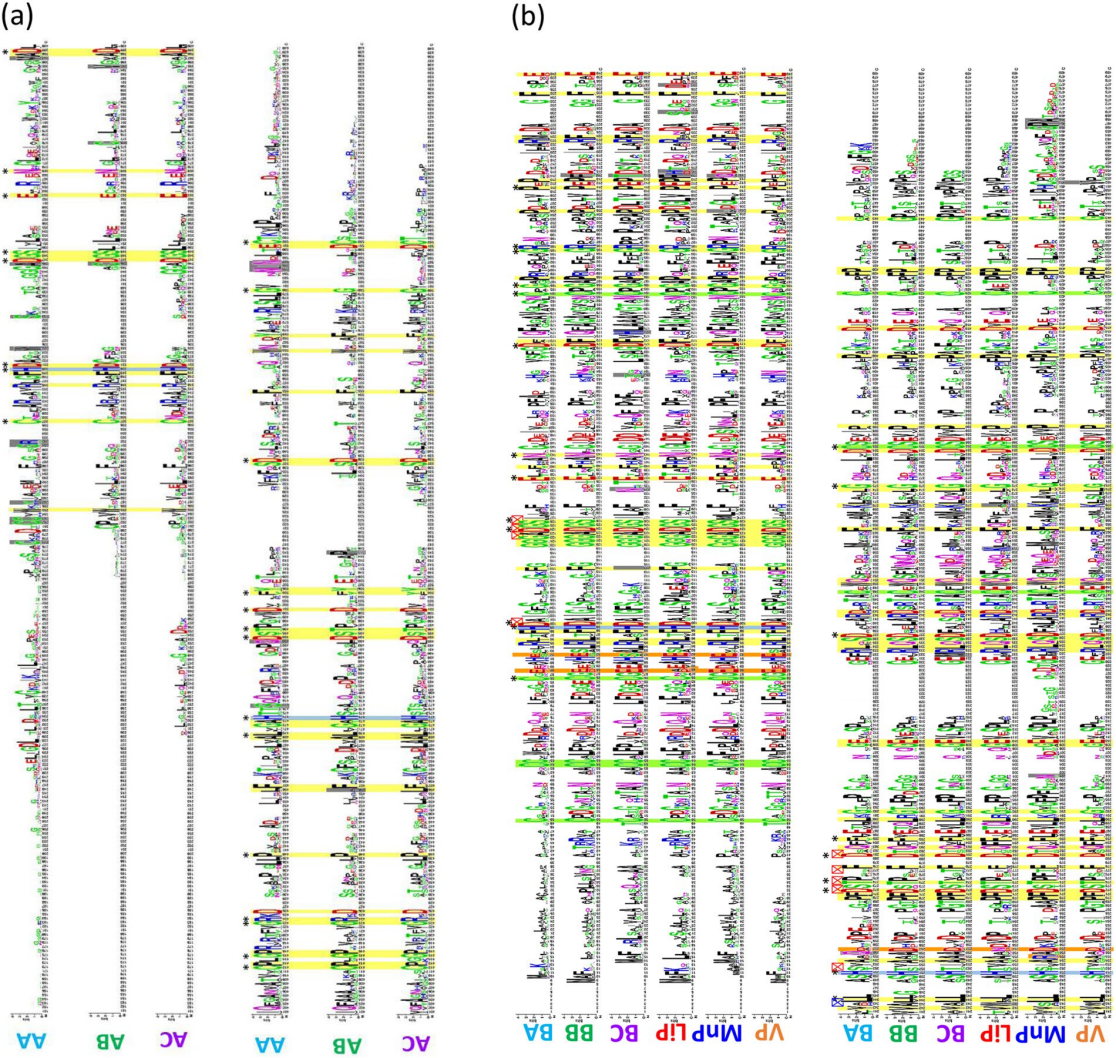
Weblogo of different CII Prxs from ascomycetes (**a**) and basidiomycetes (**b**). basidiomycete and ascomycete CII Prxs were aligned with MAFFT, and then separated into sub-classes. Weblogos were created for each group with Weblogo3 and aligned manually with the others in order to easily identify the conserved AA between the sub-classes highlighted in yellow and those that are specific to one or several sub-classes are highlighted in others colors. AA, AB and AC stand respectively for ascomycete CII Prxs sub-class A, B, and C; BA, BB and BC stand respectively for basidiomycete CII Prxs sub-class A,, B and C,, VP: Versatile peroxidases, MnP: Manganese peroxidases, LiP: Lignin peroxidases. Green highlight: eight cysteines forming four disulfide bridges; blue highlight: two active site histidines; orange highlight: three acidic residues forming the Mn^2+^ oxidation site; red dot: nine ligands of two structural Ca^2+^ ions; blue dot: one tryptophan responsible for aromatic substrate oxidation by LiP; dark gray: position specific to one class. *Conserved residues between basidiomycete and ascomycete CII Prx classes.

A high quality of annotation is mandatory to perform a global analysis of multigene families evolution such as those of the CII Prxs^44^. A set of 150 genomes from ascomycetes, basidiomycetes and early diverging fungi (Table 1) has been carefully annotated for CII Prx encoding sequences and used for phylogeny, clustering analysis and profile design. No ligninase-like sequence has been detected in any early diverging fungi analyzed. The CII Prx numbers and gene structures are highly variable. Between 1 and 15 isoforms can be detected per species and may contain up to 15 introns in a single sequence, with short exons and introns (e.g. 6 nt for the last exon). Characteristic residues necessary for haem binding and electron transport were looked for in all CII Prxs analyzed (Fig. 1). Well conserved clusters have been identified by phylogenetic analysis and the corresponding sequences were used to update the existing profiles and to construct new profiles and sub-profiles. When available, the gene structure (introns/exons) was also used to support the tree topology. This procedure, combining phylogenetic analysis, presence of key residues, gene structure analysis and construction of HMM profiles, allowed identifying mis-classifications (false positives and false negatives) and reassigning them to their appropriate sub-classes.

**Table 1:** number of sequences for Ascomycete and Basidiomycete species.

	phylum	order	Trophism	CcP	MnP	LiP	VP	CIIBA	CIIBB	CIIBC	CIIAA	CIIAB	CIIAC	Total
*Gonapodya prolifera*	Monoblepharid-omycota	Monoblepharidales	S	8	0	0	0	0	0	0	0	0	0	**0**
*Mortierella elongata*	Mortierellomycotina	Mortierellales	END/S	1	0	0	0	0	0	0	0	0	0	**0**
*Mortierella verticillata*	Mortierellomycotina	Mortierellales	END/S	2	0	0	0	0	0	0	0	0	0	**0**
*Umbelopsis ramanniana*	Mucoromycotina	Umbelopsidales	END/S	2	0	0	0	0	0	0	0	0	0	**0**
*Phycomyces blakesleeanus*	Mucoromycotina	Mucorales	S	2	0	0	0	0	0	0	0	0	0	**0**
*Mucor circinelloides*	Mucoromycotina	Mucorales	S/OP	4	0	0	0	0	0	0	0	0	0	**0**
*Rhizopus delemar*	Mucoromycotina	Mucorales		3	0	0	0	0	0	0	0	0	0	**0**
*Piromyces sp*	Neocallimastigo-mycota	Neocallimastigales	Sy	0	0	0	0	0	0	0	0	0	0	**0**
*Aspergillus fumigatus*	Pezizomycotina	Eurotiales	AP/OP/S	2	0	0	0	0	0	0	0	0	0	**0**
*Aureobasidium pullulans*	Pezizomycotina	Dothideales		2	0	0	0	0	0	0	1	0	2	**3**
*Metarhizium acridum*	Pezizomycotina	Hypocreales	APE	1	0	0	0	0	0	0	1	0	0	**1**
*Ascosphaera apis*	Pezizomycotina	Onygenales		2	0	0	0	0	0	0	0	0	0	**0**
*Penicillium marneffei*	Pezizomycotina	Eurotiales	APM	2	0	0	0	0	0	0	0	0	0	**0**
*Geomyces destructans*	Pezizomycotina	Leotiomycetes Incertae		2	0	0	0	0	0	0	0	0	0	**0**
*Ajellomyces capsulatus*	Pezizomycotina	Onygenales		2	0	0	0	0	0	0	0	0	0	**0**
*Arthroderma benhamiae ana*.	Pezizomycotina	Onygenales		2	0	0	0	0	0	0	0	0	0	**0**
*Coccidioides immitis*	Pezizomycotina	Onygenales		2	0	0	0	0	0	0	0	0	0	**0**
*Coccidioides posadasii*	Pezizomycotina	Onygenales		2	0	0	0	0	0	0	0	0	0	**0**
*Paracoccidioides brasiliensis*	Pezizomycotina	Onygenales		2	0	0	0	0	0	0	0	0	0	**0**
*Trichophyton equinum*	Pezizomycotina	Onygenales		2	0	0	0	0	0	0	0	0	0	**0**
*Trichophyton rubrum*	Pezizomycotina	Onygenales		2	0	0	0	0	0	0	0	0	0	**0**
*Trichophyton tonsurans*	Pezizomycotina	Onygenales		2	0	0	0	0	0	0	0	0	0	**0**
*Trichophyton verrucosum*	Pezizomycotina	Onygenales		2	0	0	0	0	0	0	0	0	0	**0**
*Blumeria graminis*	Pezizomycotina	Erysiphales	BPP/END	2	0	0	0	0	0	0	0	0	0	**0**
*Erysiphe pisi*	Pezizomycotina	Erysiphales		2	0	0	0	0	0	0	0	0	0	**0**
*Epichloe festucae*	Pezizomycotina	Hypocreales		1	0	0	0	0	0	0	0	0	0	**0**
*Cercospora zeae-maydis*	Pezizomycotina	Capnodiales	HPP	1	0	0	0	0	0	0	0	0	1	**1**
*Mycosphaerella graminicola*	Pezizomycotina	Capnodiales		0	0	0	0	0	0	0	1	0	1	**2**
*Colletotrichum graminicola*	Pezizomycotina	Glomerellales		1	0	0	0	0	0	0	5	1	1	**7**
*Magnaporthe grisea*	Pezizomycotina	Magnaporthales		2	0	0	0	0	0	0	2	1	0	**3**
*Cochliobolus sativus*	Pezizomycotina	Pleosporales		1	0	0	0	0	0	0	1	3	1	**5**
*Leptosphaeria maculans*	Pezizomycotina	Pleosporales		1	0	0	0	0	0	0	1	2	1	**4**
*Setosphaeria turcica*	Pezizomycotina	Pleosporales		1	0	0	0	0	0	0	1	2	1	**4**
*Melanconium sp*.	Mitosporic Ascomycota	mitosporic	NPP	2	0	0	0	0	0	0	3	1	0	**4**
*Aplosporella prunicola*	Pezizomycotina	Botryosphaeriales		2	0	0	0	0	0	0	0	1	0	**1**
*Botryosphaeria dothidea*	Pezizomycotina	Botryosphaeriales		2	0	0	0	0	0	0	1	1	2	**4**
*Cryphonectria parasitica*	Pezizomycotina	Diaporthales		2	0	0	0	0	0	0	0	2	0	**2**
*Verticillium dahliae*	Pezizomycotina	Glomerellales		1	0	0	0	0	0	0	3	0	0	**3**
*Botrytis cinerea*	Pezizomycotina	Helotiales		2	0	0	0	0	0	0	0	0	0	**0**
*Sclerotinia sclerotiorum*	Pezizomycotina	Helotiales		2	0	0	0	0	0	0	0	0	0	**0**
*Fusarium oxysporum*	Pezizomycotina	Hypocreales		2	0	0	0	0	0	0	1	0	0	**1**
*Gibberella zeae ana*.	Pezizomycotina	Hypocreales		2	0	0	0	0	0	0	1	0	0	**1**
*Nectria haematococca ana*.	Pezizomycotina	Hypocreales		1	0	0	0	0	0	0	1	0	0	**1**
*Gaeumannomyces graminis*	Pezizomycotina	Magnaporthales		1	0	0	0	0	0	0	2	0	1	**3**
*Alternaria brassicicola*	Pezizomycotina	Pleosporales		1	0	0	0	0	0	0	1	2	1	**4**
*Cochliobolus heterostrophus*	Pezizomycotina	Pleosporales		1	0	0	0	0	0	0	1	3	1	**5**
*Didymella exigua*	Pezizomycotina	Pleosporales		1	0	0	0	0	0	0	2	2	2	**6**
*Parastagonospora nodorum*	Pezizomycotina	Pleosporales		1	0	0	0	0	0	0	2	2	1	**5**
*Pyrenophora tritici-repentis*	Pezizomycotina	Pleosporales		1	0	0	0	0	0	0	1	2	1	**4**
*Cenococcum geophilum*	Pezizomycotina	mitosporic	Sy (ECM, ERM, Li)	2	0	0	0	0	0	0	0	1	0	**1**
*Meliniomyces bicolor*	Pezizomycotina	Helotiales		2	0	0	0	0	0	0	1	0	0	**1**
*Meliniomyces variabilis*	Pezizomycotina	Helotiales		2	0	0	0	0	0	0	1	0	0	**1**
*Cladonia grayi*	Pezizomycotina	Lecanorales		1	0	0	0	0	0	0	0	0	0	**0**
*Oidiodendron maius*	Pezizomycotina	Leotiomycetes incertae		2	0	0	0	0	0	0	0	0	0	**0**
*Grosmannia clavigera*	Pezizomycotina	Ophiostomatales		1	0	0	0	0	0	0	0	1	0	**1**
*Tuber melanosporum*	Pezizomycotina	Pezizales		2	0	0	0	0	0	0	0	0	0	**0**
*Xanthoria parietina*	Pezizomycotina	Teloschistales		1	0	0	0	0	0	0	0	0	0	**0**
*Daldinia eschscholzii*	Pezizomycotina	Xylariales	END	2	0	0	0	0	0	0	0	1	0	**1**
*Hypoxylon sp. C14A*	Pezizomycotina	Xylariales		2	0	0	0	0	0	0	1	1	0	**2**
*Aspergillus clavatus*	Pezizomycotina	Eurotiales	END/S	2	0	0	0	0	0	0	0	0	0	**0**
*Trichoderma asperellum*	Pezizomycotina	Hypocreales		1	0	0	0	0	0	0	0	0	0	**0**
*Baudoinia compniacensis*	Pezizomycotina	Capnodiales	S	1	0	0	0	0	0	0	0	0	1	**1**
*Zasmidium cellare*	Pezizomycotina	Capnodiales		2	0	0	0	0	0	0	0	0	3	**3**
*Aspergillus aculeatus*	Pezizomycotina	Eurotiales		2	0	0	0	0	0	0	0	0	0	**0**
*Aspergillus wentii*	Pezizomycotina	Eurotiales		2	0	0	0	0	0	0	1	0	0	**1**
*Emericella nidulans*	Pezizomycotina	Eurotiales		2	0	0	0	0	0	0	0	0	0	**0**
*Monascus ruber*	Pezizomycotina	Eurotiales		2	0	0	0	0	0	0	0	0	0	**0**
*Penicillium chrysogenum*	Pezizomycotina	Eurotiales		2	0	0	0	0	0	0	0	0	0	**0**
*Talaromyces stipitatus*	Pezizomycotina	Eurotiales		2	0	0	0	0	0	0	0	0	0	**0**
*Thermoascus aurantiacus*	Pezizomycotina	Eurotiales		2	0	0	0	0	0	0	0	0	0	**0**
*Acremonium alcalophilum*	Pezizomycotina	Glomerellales		1	0	0	0	0	0	0	1	0	0	**1**
*Trichoderma reesei ana*	Pezizomycotina	Hypocreales		1	0	0	0	0	0	0	0	0	0	**0**
*Hysterium pulicare*	Pezizomycotina	Hysteriales		2	0	0	0	0	0	0	1	1	0	**2**
*Amorphotheca resinae*	Pezizomycotina	Leotiomycetes incertae		2	0	0	0	0	0	0	0	0	0	**0**
*Uncinocarpus reesii*	Pezizomycotina	Onygenales		2	0	0	0	0	0	0	0	0	0	**0**
*Ascobolus immersus*	Pezizomycotina	Pezizales		1	0	0	0	0	0	0	0	0	0	**0**
*Cucurbitaria berberidis*	Pezizomycotina	Pleosporales		1	0	0	0	0	0	0	1	1	0	**2**
*Lentithecium fluviatile*	Pezizomycotina	Pleosporales		1	0	0	0	0	0	0	3	1	3	**7**
*Chaetomium thermophilum*	Pezizomycotina	Sordariales		1	0	0	0	0	0	0	0	0	0	**0**
*Neurospora crassa*	Pezizomycotina	Sordariales		1	0	0	0	0	0	0	0	0	0	**0**
*Podospora anserina*	Pezizomycotina	Sordariales		1	0	0	0	0	0	0	1	0	0	**1**
*Sordaria macrospora*	Pezizomycotina	Sordariales		1	0	0	0	0	0	0	0	0	0	**0**
*Sporotrichum thermophile*	Pezizomycotina	Sordariales		1	0	0	0	0	0	0	1	0	0	**1**
*Thielavia terrestris*	Pezizomycotina	Sordariales		1	0	0	0	0	0	0	0	0	0	**0**
*Apiospora montagnei*	Pezizomycotina	Xylariales		2	0	0	0	0	0	0	1	1	0	**2**
*Aspergillus flavus*	Pezizomycotina	Eurotiales	S/OP	2	0	0	0	0	0	0	0	0	0	**0**
*Aspergillus niger*	Pezizomycotina	Eurotiales		2	0	0	0	0	0	0	0	0	0	**0**
*Aspergillus oryzae*	Pezizomycotina	Eurotiales		2	0	0	0	0	0	0	0	0	0	**0**
*Neosartorya fischeri*	Pezizomycotina	Eurotiales		2	0	0	0	0	0	0	0	0	0	**0**
*Rhytidhysteron rufulum*	Pezizomycotina	Hysteriales		2	0	0	0	0	0	0	1	2	0	**3**
*Chaetomium globosum*	Pezizomycotina	Sordariales		1	0	0	0	0	0	0	0	0	0	**0**
*Trichoderma atroviride*	Pezizomycotina	Hypocreales	S/P	1	0	0	0	0	0	0	0	0	0	**0**
*Trichoderma harzianum*	Pezizomycotina	Hypocreales		1	0	0	0	0	0	0	0	0	0	**0**
*Trichoderma virens*	Pezizomycotina	Hypocreales		1	0	0	0	0	0	0	0	0	0	**0**
*Cryptococcus gattii*	Basidiomycota	Tremellales	APM	2	0	0	0	0	0	0	0	0	0	**0**
*Cryptococcus neoformans*	Basidiomycota	Tremellales		2	0	0	0	0	0	0	0	0	0	**0**
*Moniliophthora perniciosa*	Basidiomycota	Agaricales	HPP	1	0	0	0	1	0	0	0	0	0	**1**
*Amanita muscaria*	Basidiomycota	Agaricales	Sy (ECM)	1	0	0	0	0	0	0	0	0	0	**0**
*Hebeloma cylindrosporum*	Basidiomycota	Agaricales		1	0	0	0	0	0	3	0	0	0	**3**
*Laccaria bicolor*	Basidiomycota	Agaricales		1	0	0	0	0	0	1	0	0	0	**1**
*Laccaria amethystina*	Basidiomycota	Agaricales		1	0	0	0	0	0	1	0	0	0	**1**
*Piloderma croceum*	Basidiomycota	Atheliales		1	0	0	0	1	0	0	0	0	0	**1**
*Boletus edulis*	Basidiomycota	Boletales		1	0	0	0	0	0	0	0	0	0	**0**
*Paxillus involutus*	Basidiomycota	Boletales		1	0	0	0	0	0	0	0	0	0	**0**
*Paxillus rubicundulus*	Basidiomycota	Boletales		1	0	0	0	0	0	0	0	0	0	**0**
*Suillus brevipes*	Basidiomycota	Boletales		1	0	0	0	0	0	0	0	0	0	**0**
*Suillus luteus*	Basidiomycota	Boletales		1	0	0	0	0	0	0	0	0	0	**0**
*Sebacina vermifera*	Basidiomycota	Sebacinales	Sy (OMF/ECM)	2	0	0	0	0	0	0	0	0	0	**0**
*Piriformospora indica*	Basidiomycota	Sebacinales	END	1	0	0	0	0	0	0	0	0	0	**0**
*Galerina marginata*	Basidiomycota	Agaricales	S (WR)	1	0	0	0	0	6	16	0	0	0	**22**
*Gymnopus luxurians*	Basidiomycota	Agaricales		1	3	0	0	1	1	0	0	0	0	**5**
*Hypholoma sublateritium*	Basidiomycota	Agaricales		1	0	0	0	0	10	4	0	0	0	**14**
*Pleurotus ostreatus*	Basidiomycota	Agaricales		1	0	0	6	3	0	0	0	0	0	**9**
*Plicaturopsis crispa*	Basidiomycota	Amylocorticiales		1	0	0	1	2	4	0	0	0	0	**7**
*Auricularia delicata*	Basidiomycota	Auriculariales		1	0	0	0	17	1	0	0	0	0	**18**
*Punctularia strigosozonata*	Basidiomycota	Corticiales		1	9	0	0	0	2	0	0	0	0	**11**
*Sphaerobolus stellatus*	Basidiomycota	Geastrales		1	20	0	2	0	2	0	0	0	0	**24**
*Bjerkandera adusta*	Basidiomycota	Polyporales		1	12	0	0	2	5	0	0	0	0	**19**
*Ceriporiopsis subvermispora*	Basidiomycota	Polyporales		1	12	2	0	1	1	0	0	0	0	**16**
*Cerrena unicolor*	Basidiomycota	Polyporales		1	0	3	0	4	9	0	0	0	0	**16**
*Dichomitus squalens*	Basidiomycota	Polyporales		1	4	0	3	3	2	0	0	0	0	**12**
*Ganoderma sp*	Basidiomycota	Polyporales		1	0	0	1	4	3	0	0	0	0	**8**
*Phanerochaete carnosa*	Basidiomycota	Polyporales		1	7	4	0	0	0	0	0	0	0	**11**
*Phanerochaete chrysosporium*	Basidiomycota	Polyporales		1	7	10	0	1	0	0	0	0	0	**18**
*Phlebia brevispora*	Basidiomycota	Polyporales		1	6	5	0	0	3	0	0	0	0	**14**
*Phlebiopsis gigantea*	Basidiomycota	Polyporales		1	6	3	0	1	0	0	0	0	0	**10**
*Polyporus arcularius*	Basidiomycota	Polyporales		1	0	6	2	6	5	0	0	0	0	**19**
*Trametes versicolor*	Basidiomycota	Polyporales		1	0	11	2	2	9	0	0	0	0	**24**
*Heterobasidion annosum*	Basidiomycota	Russulales		1	3	0	0	2	5	0	0	0	0	**10**
*Stereum hirsutum*	Basidiomycota	Russulales		1	0	0	0	1	0	5	0	0	0	**6**
*Tremella mesenterica*	Basidiomycota	Tremellales		2	0	0	0	0	0	0	0	0	0	**0**
*Schizophyllum commune*	Basidiomycota	Agaricales	S (DND)	1	0	0	0	0	0	0	0	0	0	**0**
*Jaapia argillacea*	Basidiomycota	Jaapiales		1	0	0	0	1	0	0	0	0	0	**1**
*Fistulina hepatica*	Basidiomycota	Agaricales	S (BR)	1	0	0	0	0	0	0	0	0	0	**0**
*Coniophora puteana*	Basidiomycota	Boletales		1	0	0	0	0	0	0	0	0	0	**0**
*Hydnomerulius pinastri*	Basidiomycota	Boletales		1	0	0	0	0	0	0	0	0	0	**0**
*Serpula lacrymans*	Basidiomycota	Boletales		1	0	0	0	0	0	0	0	0	0	**0**
*Botryobasidium botryosum*	Basidiomycota	Cantharellales		1	0	0	0	0	0	0	0	0	0	**0**
*Dacryopinax sp*	Basidiomycota	Dacrymycetales		1	0	0	0	0	0	0	0	0	0	**0**
*Gloeophyllum trabeum*	Basidiomycota	Gloeophyllales		1	0	0	0	0	0	0	0	0	0	**0**
*Neolentinus lepideus*	Basidiomycota	Gloeophyllales		1	0	0	0	0	0	0	0	0	0	**0**
*Daedalea quercina*	Basidiomycota	Polyporales		1	0	0	0	0	1	0	0	0	0	**1**
*Fomitopsis pinicola*	Basidiomycota	Polyporales		1	0	0	0	1	0	0	0	0	0	**1**
*Laetiporus sulphureus*	Basidiomycota	Polyporales		1	0	0	0	1	0	0	0	0	0	**1**
*Postia placenta*	Basidiomycota	Polyporales		1	0	0	0	1	0	0	0	0	0	**1**
*Wolfiporia cocos*	Basidiomycota	Polyporales		1	0	0	0	1	0	0	0	0	0	**1**
*Agaricus bisporus*	Basidiomycota	Agaricales	Soil/litter decomposer	1	0	0	2	0	0	0	0	0	0	**2**
*Amanita thiersii*	Basidiomycota	Agaricales		1	0	0	0	0	0	0	0	0	0	**0**
*Coprinopsis cinerea*	Basidiomycota	Agaricales		1	0	0	0	1	0	0	0	0	0	**1**
*Dioszegia cryoxerica*	Basidiomycota	Tremellales		4	0	0	0	0	0	0	0	0	0	**0**

S: Saprotrophe (Wood Decay: BR = Brown rot; WR = White rot; DND = Decay chemistry Not Defined); PP: Plant Pathogen (NPP: Necrotrophic PP; BPP: Biothophic PP; HPP: Hemibiotrophic PP; OP: Opportunistic Pathogen); AP: Animal Pathogen (APM: Animal Pathogen on Mammals; APE: Animal Pathogen Entomopathogenic); Sy: Symbiotic (ECM: Ectomycorrhizal fungus; ERM: Ericoid Mycorrhizal fungus; OMF: Orchid Mycorrhizal fungus; Li: Lichen); END: endophytic fungus; U:Unicell; Mycel: Mycelium; D: Dimorphic species; T: thallus with rhizoids.

**Figure 2:**
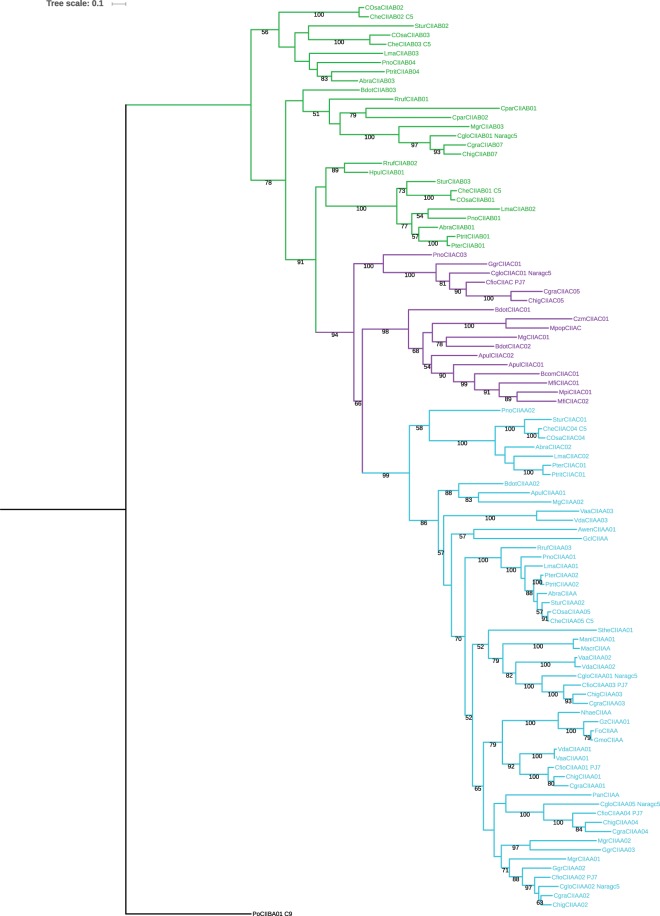
Phylogenetic tree of ascomycetes CII Prx sequences. 99 sequences coming from 26 ascomycetes have been aligned to generate the tree. A basidiomycete sequence was used as outgroup. CIIAA are represented in blue, CIIAB in green and CIIAC in purple. Bootstrap values higher than 50% are indicated.

Sequences detected in ascomycetes form a group distinct from the well-described basidiomycete ligninases (Supplementary Fig. S1). The clear distribution of ascomycete ligninases in three clusters (Fig. 2) helps defining and designing three sub-classes of ascomycete ligninases, thereafter referred as ascomycete sub-class A (CIIAA), sub-class B (CIIAB) and sub-class C (CIIAC), and their corresponding profiles (Fig. 1a). By comparing the conserved residues found in basidiomycetes and ascomycetes (Fig. 1), we can see that (i) many residues dispersed throughout the sequences are conserved (~20 aa), between these two very distant phyla; (ii) the 3 sub-classes defined in ascomycetes do not share all of the defined residues with catalytic properties found in basidiomycetes, and miss most of the cysteine residues responsible for protein stability; (iii) the ascomycete CIIAA sub-class has the more divergent sequence profile, which is consistent with its phylogenetic position (Fig. 2). Gene structure analysis doesn’t reveal conserved common introns between the three sub-classes. Extensive changes in the exon-intron structure (intron gain and loss) have already been described for members of the *Fusarium* clade and appear to be the normality in ascomycetes^45^.

Members of these 3 new sub-classes are only detected in Pezizomycotina and are absent from the other ascomycete sub-phyla. Moreover, within the Pezizomycotina sub-phylum, ligninase encoding sequences are not detected in all species. They are mainly found in species known to interact with plants: pathogenic ascomycetes, either necrotrophic or hemibiotrophic, possess up to 7 sequences, whereas few or no sequence were detected in saprotrophic species (Table 1).

**Figure 3:**
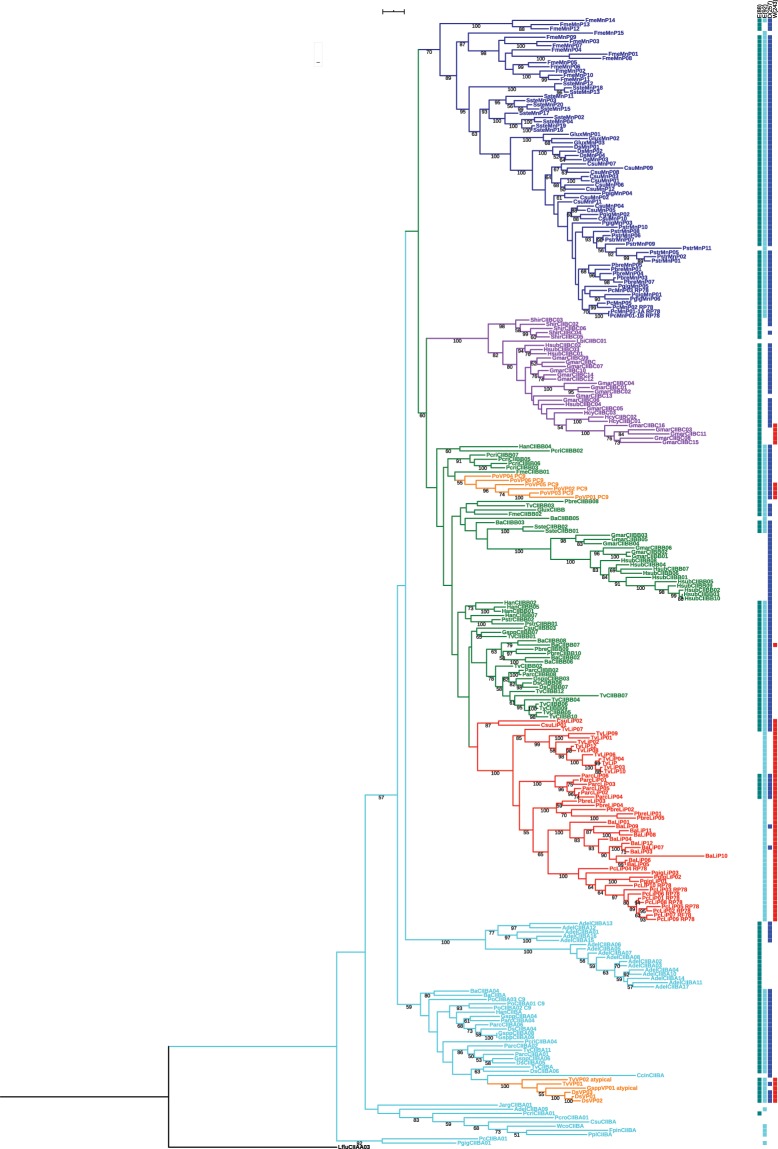
Phylogenetic tree of basidiomycetes CII Prx sequences. 267 sequences coming from 28 basidiomycetes have been aligned to generate the tree. One Ascomycete sequence was used as outgroup. LiP sequences are represented in red, VP in orange, CIIBB in green, CIIBC in pink, MnP in blue and CIIBA in azure. Bootstrap values higher than 50% are indicated. Presence/absence of 3 conserved residues (E(35), E(39) and D(179)) responsible for Mn^2+^ oxidation in MnP, as well as the catalytic W typical from LiP are displayed aside, respectively with green, azure, blue and red square.

The situation for basidiomycetes is much more complex. The exhaustive mining of 68 basidiomycete genomes has demonstrated the need to redefine the existing profiles and highlighted that some sequences do not belong to the four previously identified groups (LiP, MnP, VP, GP). Three new basidiomycete ligninase sub-classes have been defined, basidiomycete sub-class A (CIIBA), sub-class B (CIIBB) and sub-class C (CIIBC) (Fig. 3). The definition of these 3 new sub-classes also led to re-affect some sequences previously known as MnP or VP. Notably, most sequences of our CII BB class were previously attributed to the MnP-class (mostly short-MnPs), but the phylogenetic analysis suggests a more restricted definition of the MnP class. The analysis of our phylogenetic tree together with the conservation of the catalytic tryptophan (conserved in LiP and VP) and Mn^2+^ oxidation sites (in MnP and VP) (Fig. 3) clearly show that the sub-classes cannot be resumed to the presence/absence of key residues. It is noteworthy that sequences possessing all the pointed residues and thus susceptible to be VP sequences are scattered in the branches of the CIIBB and CIIBA sub-classes. Interestingly such sequences are also present at the basis of the LiP clade, with two sequences from *C. subvermispora*, described as phylogenetically and catalytically intermediate between classical LiPs and VPs^8^. Few specific residue conservations can be detected in each sub-class (Fig. 1b), but the CIIBC sub-class is apparently the more divergent one.

**Figure 4:**
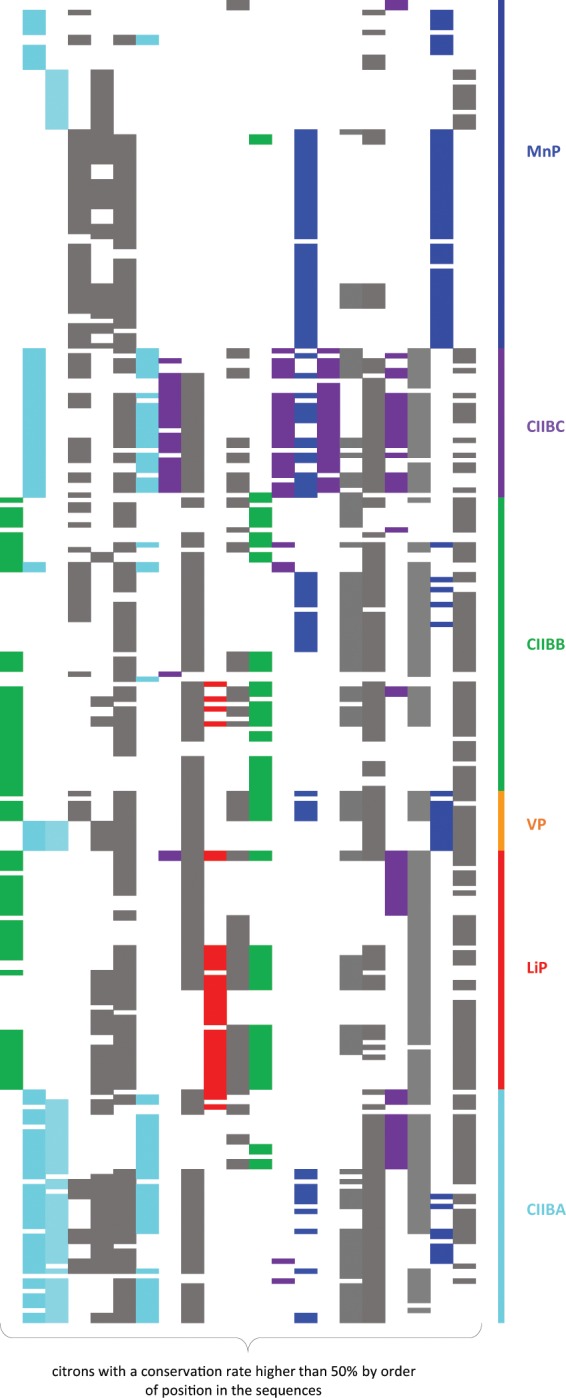
Conserved common introns from 267 basidiomycete sequences belonging to 28 organisms. Cintrons were extracted from CIWOG’s database and 21 out of 55 were considered conserved since they were present in one or several sub-classes with a conservation rate higher than 50%. Cintrons are highlighted in accordance with the class in which they are in majority. Cintrons common to all classes are highlighted in grey.

Phylogenetic and profile analysis were mostly supported by gene structure (intron number and position) conservation in basidiomycetes. Out of 57 common introns (cintrons) detected with CIWOG, 21 were considered conserved since they were present in one or several sub-classes with a conservation rate higher than 50% (Fig. 4). Only seven introns are found in sequences from all families. The family with the more noteworthy pattern of intron composition is the MnP: 3 introns are specific to MnP sequences, and 2 others are only shared with CIIBA sub-class, whereas 3 introns are present in all classes except MnP. The CIIBC sub-class also possesses one intron totally specific and two others very marginal elsewhere. Three introns are mostly common to CIIBB and LiP, which confirms the phylogenetic hypothesis that LiP sequences originate from CIIBB. As to the VP sequences, they are rather intron-rich, with some sequences resembling the CIIBB introns’ pattern, and some others the CIIBA one.

**Figure 5:**
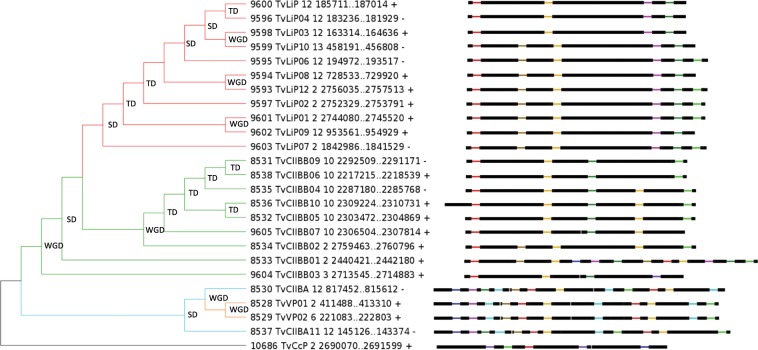
Phylogenetic analysis, gene localisation, orientation and structure of 24 CII Prxs sequences from *Trametes versicolor*. Gene structures produced with GECA have been included in front of each branch. LiP sequences are represented in red, CIIBB in green and CIIBA in azure. Tandem duplication (TD), segmental duplication (SD) and whole genome duplication (WGD) events are represented on the base of the branch they occur on. Name in front of each branch contains ID and name attributed in the RedoxiBase, the scaffold number, gene coordinates and orientation.

In basidiomycetes, extensive recent gene duplications in LiP, MnP and the three other new sub-classes were identified. The 24 CII Prx sequences of *Trametes versicolor* distributed among LiP, VP, CIIBA and CIIBB are mainly clustered in 3 genomic regions and are the result of tandem (TD), segmental (SD) and whole genome duplications (WGD) (Fig. 5). These events have been defined as following: TD as successive duplicated genes, SD as blocks of DNA that map to different loci in the same chromosome and WGD as blocks of DNA that map different chromosomes. However, all these duplications are very recent since they form well-supported clusters specific for each species (Fig. 3). It begs the question of duplication events widespread among basidiomycetes but it appears that other peroxidase families such as Cytochrome C peroxidases (CcP) or glutathione peroxidases were less or not subjected to duplication. Besides, no correlation can be made with the distribution of transposable elements. This suggests that these duplications are probably an evolutionary response to selection pressure.

### Working hypothesis of ligninase evolution

Ligninases are detected in ascomycetes and basidiomycetes but are absent from the early diverging fungi analyzed (*Choanephora cucurbitarum*, *Mucor circinelloides*, *Phycomyces blakesleeanus*, *Rhizopus oryzae*). These two phyla are monophyletic sister groups belonging to Dikarya which emerged from a common ancestral organism. Key residues described in basidiomycete MnP, LiP, and VP are also detected in CII Prx ascomycete sequences (Fig. 1). This suggests that CII Prxs detected in ascomycetes and basidiomycetes could have evolved independently from a similar ancestral sequence following convergent evolution. Notably, CII Prxs belong to the same superfamily as the class I Cytochrome C peroxidases (CcP), and share common key residues^23^. The taxonomic distribution of the CI Prxs was clarified in order to enable a better understanding of the overall CII Prxs evolution^46^. CI Prxs are found in plants, fungi, and prokaryotes. Unlike CII and CIII Prxs, they are not glycosylated and do not have signal peptides, calcium ions, or disulfide bridges. They contain five main groups of proteins: (i) Catalase peroxidases (CP) present in prokaryotes and in some eukaryotes following a gene transfer, (ii) Cytochrome c peroxidases (CcP) found in mitochondria containing organisms but not detectable in Viridiplantae, (iii) Ascorbate peroxidases (APx) found only in chloroplastic organisms, and (iv)(v) two hybrid-type peroxidases detected in fungi and different kingdoms. A previous phylogenetic study suggested evolutionary relation between CI Prxs and CII Prxs^47^.

When comparing ascomycetes and basidiomycetes for the presence of CcP and ligninase encoding sequences (Table 1 and Supplementary Table S1), we can observe that: (i) in most cases, two CcP sequences are detected in fungi which do not possess CII Prx sequences and (ii) only one CcP sequence is detected when the genome contains at least one CII Prx sequence. This suggests that CII Prxs would have emerged from an ancestral sequence that could be a CcP sequence. A similar theory of evolution has already been described for the CIII Prxs^46^. Indeed, CIII Prxs are only detected in plants, which lack CcP. CIII Prxs and CII Prxs are both subjected to numerous species specific duplications (tandem and segmental duplications), contain highly conserved cysteine residues necessary for disulfite bridges and stability of secreted proteins. As an alternative hypothesis, all fungi would possess at least one CII Prx sequence and loss events occurred more recently. On the principle of maximum parsimony (Supplementary Table S1), this hypothesis seems unlikely since it would require many independent events of gene loss.

In addition, intron positions and numbers are not conserved between the CII Prxs found in ascomycetes and in basidiomycetes. Basidiomycetes contain more introns than ascomycetes (on average 8 and 2 respectively), but intron sizes are higher, on average, in ascomycetes (74 nt) than in basidiomycetes (54 nt). Similar phenomena regarding intron size and number are also observed with other families such as glutathione peroxidases (1 or 2 introns for ascomycetes and 3 to 6 for basidiomycetes). Altogether, these results could suggest independente evolution from two ancestral sequences containing no or few introns. This is in accordance with the hypothesis of a convergent evolution from an existing CI Prx sequence for these two lineages, after the ascomycetes/basidiomycetes separation.

Peroxidase family expansion and recent gene loss processes are likely to be both involved in the history of these genes in fungi. This recent evolutionary history seems particularly driven by fungal life style and leads to numerous adaptive convergence to environment, particularly to host immunity. Typically, necrotrophic or hemibiotrophic fungi, either ascomycetes or basidiomycetes, possess CII Prxs while symbiotic, endophytic or biotrophic fungi mostly do not. WR basidiomycetes and necrotrophic pezizomycetes (ascomycetes) present the highest levels of CII Prxs (Table 1).

Even if numerous key residues are well conserved between the different CII Prx classes, the residues described as necessary for electron transfer are missing in Ascomycetes. Furthermore, the position of the conserved cysteines varies between CII Prxs of ascomycetes and basidiomycetes. These divergences support the independent emergence of CII Prxs in ascomycetes and basidiomycetes and lead to suggest divergent functions (or different catalytic mechanisms). Lignin-degrading fungi possess large batteries of ligninase encoding sequences which enable them to oxidize the lignin polymer and then to use it as a source of carbon. Saprophytic ascomycetes, as well as BR basidiomycetes, described as not being lignivor, present none or low number of ligninase encoding sequences. They probably just use them to depolymerize the lignin by oxidation in order to increase the accessibility to other cell wall components such as cellulose and hemicellulose. Finally, plant pathogenic ascomycetes (either necrotrophic or hemibiotrophic), which contain up to 7 ligninases encoding sequences, use these proteins to depolymerize the lignin in order to access and to infect the host cell. This conclusion is in agreement with the fact that plant pathogen fungi also possess more CAZymes^48^. A more detailed analysis of the distribution into classes in basidiomycete white rot fungi reveals that most species possess only one enzyme among the 3 mains, i.e. MnP, LiP and VP, and, in our data, 3 species (*Auricularia*, *Hypholoma* and *Stereum*) possess neither of them. Phanerochaete, Phlebia, Phlebiopsis and Ceriporiopsis are the only ones that have MnP and LiP sequences together. All analyzed organisms possess at least one complementary class of ligninase among the sub-classes. This diversity of ligninolytic enzymes is probably a major key for the fungal ability to proliferate on different types of wood^2,49^.

## Conclusions

The availability of a large number of fungal genomes (1000 fungal genomes project, https://genome.jgi.doe.gov/programs/fungi/index.jsf)^50^ allows us to perform exhaustive and expert data mining for the analysis of CII Prxs. They are present in the majority of basidiomycetes, only detected in the Pezizomycotina phylum belonging to ascomycetes whereas absent in early diverging fungi. Sequences found in ascomycetes and basidiomycetes present highly divergent gene structures, and sequence conservation only for key residues scattered throughout the sequences. Altogether, this suggests that actual CII Prxs found in basidiomycetes and ascomycetes probably originate from at least two independent events after the separation between ascomycetes and basidiomycetes. This confirms the conclusion obtained for basidiomycetes with tree reconciliation^24^. In addition, the sequence similarity with the CI Prx, CcP sequence, and the correlation between lack of the second CcP copy and presence of CII Prx suggest this ancestral sequence could be a CcP.

Four residues already described in MnP, LiP and VP as necessary for electron transfer are sometimes missing in the new closely related ascomycete and basidiomycete sub-classes. The discrepancy between catalytic activity based on few residues and the global sequence conservation is not antagonistic. Indeed, numerous other residues are highly conserved between all CII and within the different sub-classes, suggesting that new CII Prxs could be able to oxidized lignin but with a different electron transfer mechanism. In all cases, the catalytic activity of CIIAA, CIIAB, CIIAC, CIIBA, CIIBB and CIIBC proteins is not yet known and needs to be demonstrated.

In basidiomycetes, the presence of MnP, LiP and VP have been clearly associated with a specific wood material decaying activity thanks to their lignin degradation capacity. CIIBA, CIIBB and CIIBC are mainly detected in wood degrading fungi (white rot), alone or associated with the main CII Prxs (MnP, LiP or VP). But they can also be found alone in brown rot fungi, plant pathogens, litter decomposing fungi and fungi with no defined decaying machinery. On the other hand, CIIAA, CIIAB and CIIAC are found in rather low copy number mainly in plant pathogens ascomycetes. The specific sequence distribution among organisms suggests that these proteins could have two separated purposes: lignin degradation as carbon source and cell penetration.

Early diverging fungi such as Chytridiomycota species are capable to degrade cellulose and pectins which allow them to used wall polymers as a carbon source^51^. But the lack of CII Prx in all early diverging fungi tested, questions about the accessibility to these carbon source for fungi that do not have the tools to degrade lignin. The absence of ligninases in early divergent fungi has raised the hypothesis of the incidence of microbial on carbon burial at the end of Paleozoic (Floudas *et al*. 2012), in opposition to a geological hypothesis^52,53^.

The following questions to address are the respective roles of these different enzymes during wood decay. This would help to better understand the biology of these fungi, and the chemical mechanisms involved in the biological decomposition of wood.

## Supplementary Information

Supplementary Information.

Supplementary Information 2.
