# Supplementary figures and images for: In silico definition of new ligninolytic peroxidase sub-classes in fungi and putative relation to fungal life style
Source: Sci Rep. 2019 Dec 30;9:20373. doi: 10.1038/s41598-019-56774-4 (PMC6937255; doi:10.1038/s41598-019-56774-4)

## Slide 1
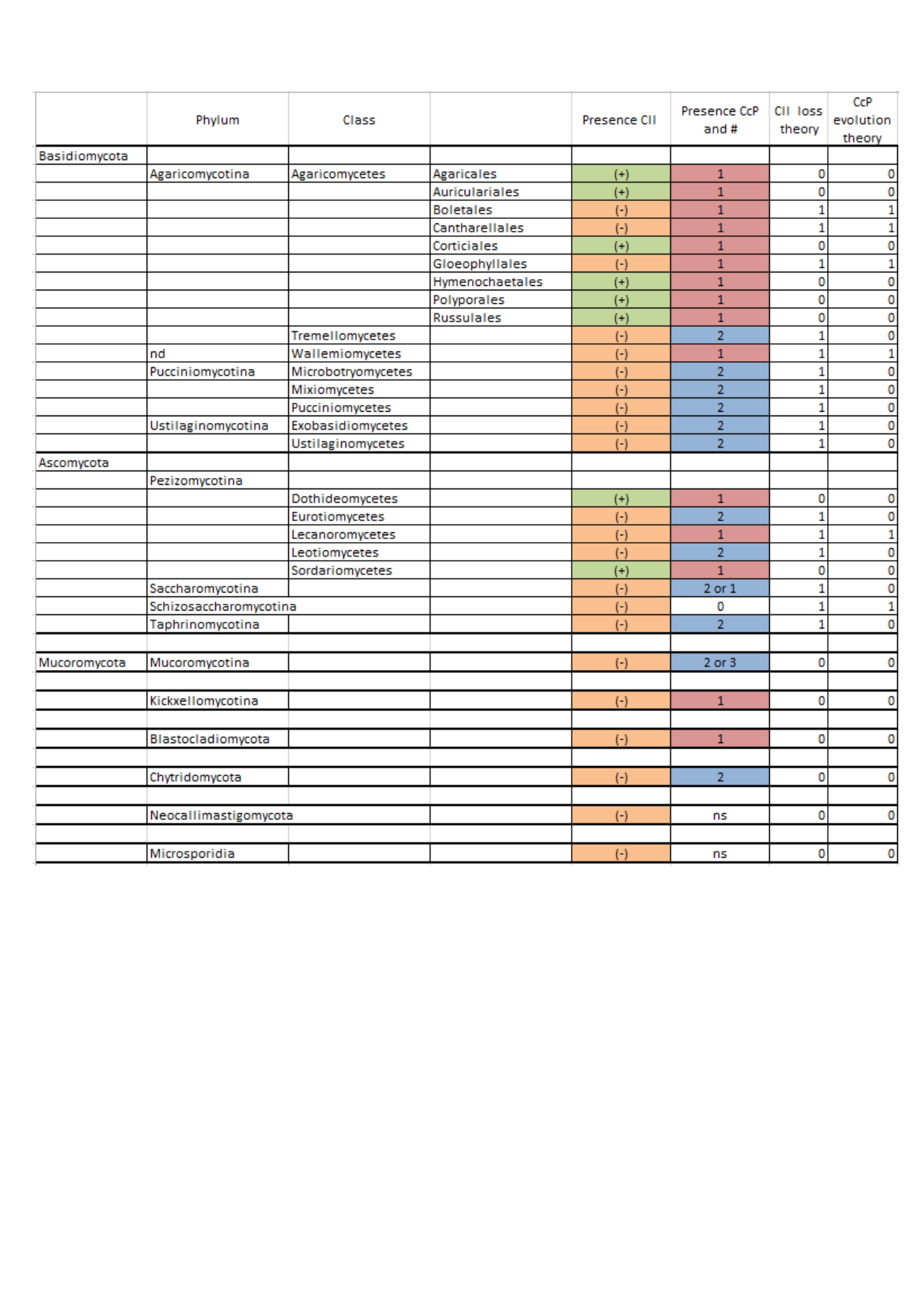

Lant

Supplement: Supplementary file 1 — Supplementary Information. [file 41598_2019_56774_MOESM1_ESM.ppt]

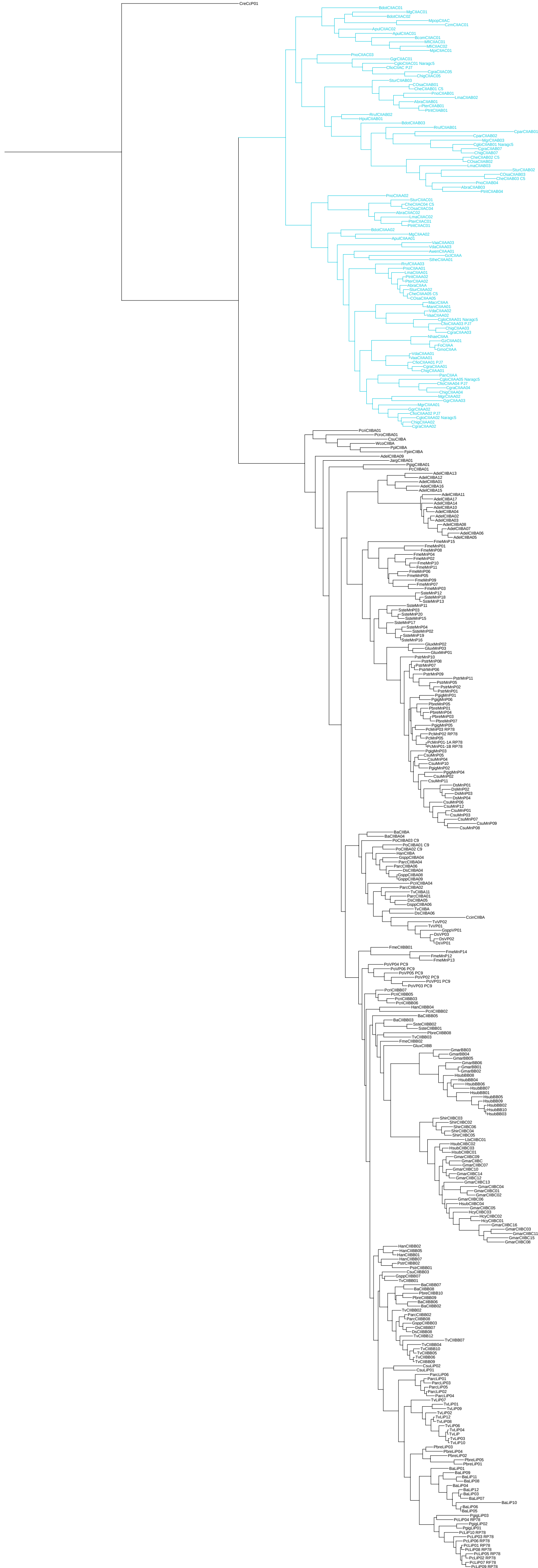

Supplement: Supplementary file 2 — Supplementary Information 2. [file 41598_2019_56774_MOESM2_ESM.pdf]
